# Anti-Inflammatory and Immunosuppressive Effects of the A_2A_ Adenosine Receptor

**DOI:** 10.1100/tsw.2011.22

**Published:** 2011-02-03

**Authors:** Gillian R. Milne, Timothy M. Palmer

**Affiliations:** Institute of Cardiovascular and Medical Sciences, College of Medical, Veterinary and Life Sciences, University of Glasgow, Scotland

**Keywords:** adenosine, A_2A_ adenosine receptor, inflammation, immunity, ischaemia-reperfusion, nuclear factor κB (NFκB), janus kinase (JAK), signal transducer and activator of transcription (STAT)

## Abstract

The production of adenosine represents a critical endogenous mechanism for regulating immune and inflammatory responses during conditions of stress, injury, or infection. Adenosine exerts predominantly protective effects through activation of four 7-transmembrane receptor subtypes termed A_1_, A_2A_, A_2B_, and A_3_, of which the A_2A_ adenosine receptor (A_2A_AR) is recognised as a major mediator of anti-inflammatory responses. The A_2A_AR is widely expressed on cells of the immune system and numerous *in vitro* studies have identified its role in suppressing key stages of the inflammatory process, including leukocyte recruitment, phagocytosis, cytokine production, and immune cell proliferation. The majority of actions produced by A_2A_AR activation appear to be mediated by cAMP, but downstream events have not yet been well characterised. In this article, we review the current evidence for the anti-inflammatory effects of the A_2A_AR in different cell types and discuss possible molecular mechanisms mediating these effects, including the potential for generalised suppression of inflammatory gene expression through inhibition of the NF-κB and JAK/STAT proinflammatory signalling pathways. We also evaluate findings from *in vivo* studies investigating the role of the A_2A_AR in different tissues in animal models of inflammatory disease and briefly discuss the potential for development of selective A_2A_AR agonists for use in the clinic to treat specific inflammatory conditions.

## INTRODUCTION

Adenosine is a ubiquitous purine nucleoside that accumulates extracellularly in response to metabolic stresses such as hypoxia and inflammation. It exerts a wide range of physiological effects via ligation of four 7-transmembrane receptor subtypes termed $A_1$, $A_{2A}$, $A_{2B}$, and $A_3$. A substantial body of evidence has demonstrated that important cell types, such as eosinophils and mast cells, express specific combinations of these receptors, and several studies have suggested that pharmacological blockade of the $A_{2B}$ and $A_3$ subtypes may be of particular benefit in attenuating the eosinophil trafficking, lung fibrosis, and chronic inflammation responsible for chronic obstructive pulmonary disease (COPD) and asthma[1].

However, most attention has been devoted to the $A_{2A}$ adenosine receptor ($A_{2A}$AR), which is expressed on most lymphoid cells, allowing it to regulate numerous aspects of the immune response, where it fulfils a predominantly anti-inflammatory role. This has been demonstrated through numerous *in vitro* and *in vivo* studies using $A_{2A}$AR-selective agonists to suppress inflammation and also by observation of enhanced inflammatory responses in $A_{2A}$AR-deficient mice[1,2,3,4]. In this article, we outline the role of the $A_{2A}$AR in a number of different cell types of the immune system and evaluate possible molecular mechanisms that may mediate its anti-inflammatory properties. We also review data obtained in studies investigating the effect of $A_{2A}$AR activation or inactivation in different tissues in animal models of inflammatory disease.

## HOW IS ADENOSINE PRODUCED *IN VIVO*?

Under normal conditions, adenosine is continuously released from cells as a product of ATP degradation. However, during conditions of stress, such as hypoxia during inflammation, levels of extracellular adenosine rise dramatically (up to 200-fold). This is partly due to increased production of AMP in hypoxic conditions, but substantial amounts of adenosine are also produced by the sequential dephosphorylation of adenine nucleotides released from platelets and haematopoietic cells, as well as damaged cells[5,6,7]. Ecto-apyrases, such as CD39, hydrolyse ATP or ADP to AMP, which is then converted to adenosine by the ecto-5'-nucleotidase CD73[8], which appears to be the rate-limiting step for adenosine production (Fig. 1). These enzymes are particularly abundant in both lymphocytes and endothelial cells, and can be induced in response to chronic hypoxia to enhance adenosine production. In addition, CD73 expression on endothelial cells can be up-regulated by IFNα[9] and adenosine itself[10]. The protective nature of this pathway has been demonstrated using gene targeting in mice. In these studies, lack of either CD39[11] or CD73[12] has been shown to result in loss of adenosine-mediated potentiation of endothelial barrier function, predominantly through the $A_{2B}$AR, leading to excessive vascular permeability and leakage following chronic hypoxia. Clearance of adenosine is achieved predominantly via its deamination to inosine by adenosine deaminase (ADA). While usually considered a cytosolic enzyme, it is now appreciated that lymphoid and dendritic cells can express an ecto-ADA population, which can be found in complexes with cell surface $A_1$ and $A_{2B}$ARs. The relatively high levels of ADA and low CD39/CD73 expression found in lymphoid cells have led to the suggestion that lymphoid cells maintain a high ATP/low adenosine environment to limit adenosine-mediated suppression of immune responses. For a comprehensive overview of the various ecto-enzymes involved in the metabolism of extracellular adenine nucleotides and adenosine, the reader is referred to an excellent recent review on the subject[8].

## $A_{2A}$AR SIGNALLING

Classically, signalling through the $A_{2A}$AR relies on its coupling to the heterotrimeric G-protein $G_s$ and stimulation of adenylyl cyclase (AC)[13]. This results in elevation of intracellular levels of cyclic AMP (cAMP), which then activate downstream effectors, including protein kinase A (PKA), cyclic nucleotide-gated ion channels, and exchange proteins directly activated by cAMP (EPACs)[14]. Opposing the actions of AC are a family of cAMP phosphodiesterases (PDEs), which hydrolyse cAMP to 5'AMP, leading to signal termination. The localisation of PDEs to specific domains within the cell leads to the generation of cAMP gradients. Critically, this allows compartmentalisation of signalling, as PKA and EPAC are anchored in specific locations in the cell by scaffold proteins, such as A-kinase anchoring proteins (AKAPs). Depending on the availability of cAMP, only a subset of these effector enzymes and their downstream substrates will be activated[15,16]. Stimulation of the $A_{2A}$AR also results in activation of the ERK signalling cascade through a number of different mechanisms that vary between cell types. For example, in CHO cells heterologously expressing the $A_{2A}$AR and in PC12 cells, $G_s$ stimulation results

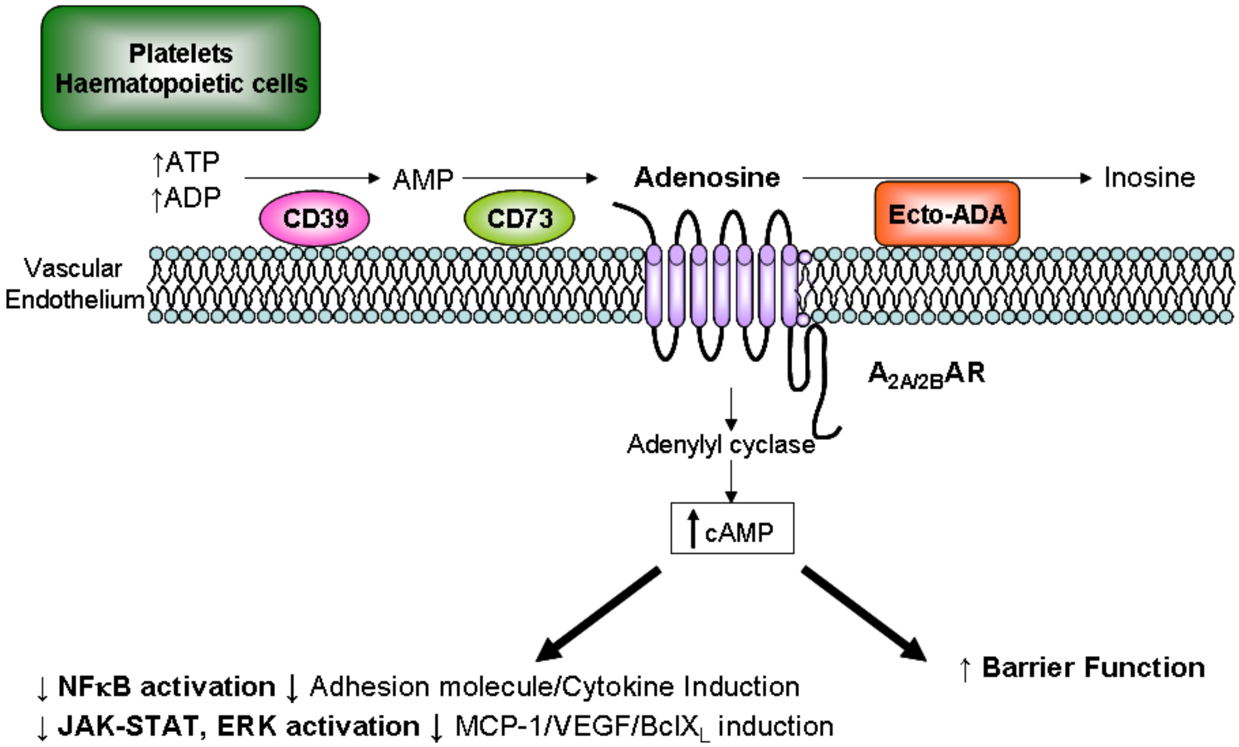

**FIGURE 1.** Interaction of extracellular adenine nucleotide metabolism with adenosine receptor signalling at the vascular endothelium. An increase in the release of ATP and ADP from endothelial cells, platelets, and haematopoietic cells under conditions of hypoxia, stress, or inflammation leads to its hydrolysis by ecto-apyrase CD39 to AMP, which is then converted to adenosine by the ecto-5'-nucleotidase CD73, both of which are abundantly expressed on the surface of the vascular endothelium. Adenosine can then bind and activate $A_{2A}$ and $A_{2B}$ARs expressed on vascular endothelial cells. While both receptors are typically expressed in endothelial cells, the $A_{2A}$AR displays a higher affinity for the endogenous agonist adenosine and will thus be activated at lower agonist concentrations than the $A_{2B}$AR. Both receptors can couple positively to adenylyl cyclase to elevate intracellular levels of cAMP. This triggers a plethora of protective effects via its intracellular sensors PKA and EPAC1: these include the inhibition of proinflammatory NFκB and JAK/STAT signalling pathways, and the enhancement of endothelial barrier function. These responses are largely transient in nature due at least in part to the ability of ecto-ADA to promote the deamination of adenosine to inosine, which is inactive at $A_{2A}$ and $A_{2B}$ARs.

in PKA-mediated activation of ERK via Src kinases[17]. Conversely, in endothelial cells and HEK293 cells, ERK can be activated independently of $G_s$ and cAMP elevation, and requires Ras[18,19]. The $A_{2A}$AR can also recruit the Arf6 guanine nucleotide exchange factor (GEF), ARNO, which is required for sustained $A_{2A}$AR-mediated activation of the ERK pathway in HEK293 cells[20].

## SUPPRESSION OF INFLAMMATORY RESPONSES *IN VITRO*

The $A_{2A}$AR is expressed on many lymphoid cells, including neutrophils, monocytes, macrophages, T cells, and natural killer (NK) cells, and its activation by adenosine or adenosine analogues results in a wide range of anti-inflammatory and immunosuppressive responses[1,2,4].

## Neutrophils

Neutrophils are one of the first cell types to be recruited to a site of injury or inflammation. They are activated by inflammatory stimuli from pathogens or damaged cells via pathogen-associated molecular pattern molecule (PAMP) receptors, such as Toll-like receptor (TLR) 4, and damage-associated molecular pattern (DAMP) receptors, such as the receptor for advanced glycation end products (RAGE), which are expressed on the neutrophil cell surface[21,22]. Activated neutrophils adhere to the endothelium and are attracted into tissues where they phagocytose and destroy pathogenic material through the release of antimicrobial proteins and proteases, and the production of a respiratory burst[23,24]. Activation of the $A_{2A}$AR on neutrophils has long been known to have a suppressive effect on their cytotoxic functions by inhibiting phagocytosis[25], production of reactive oxygen metabolites[26,27], and adherence to the endothelium[27,28]. More recent studies have shown that most of these inhibitory effects are dependent upon $A_{2A}$AR-mediated elevation of intracellular cAMP and activation of PKA. For example, the $A_{2A}$AR-selective agonist ATL193 inhibits neutrophil oxidative activity via a cAMP- and PKA-dependent mechanism[29]. $A_{2A}$AR activation also inhibits neutrophil recruitment to inflammatory sites in a PKA-dependent manner by down-regulating expression of the neutrophil adhesion molecule “very late antigen 4” (VLA-4), which interacts with vascular cell adhesion molecule-1 (VCAM-1) to enable adherence to the endothelium[27,30].

## Macrophages

Macrophages are present in all tissues and, like neutrophils, express innate immune receptors that mediate their activation during the earliest stages of an immune response. In addition, neutrophils and macrophages release cytokines and chemokines that activate the endothelium, and enable recruitment of circulating monocytes to the site of inflammation where they subsequently differentiate into macrophages. Activated macrophages phagocytose cell debris and pathogenic material, and can kill engulfed organisms either nonspecifically or through activation of specific lymphocyte responses. They also play a central role in shaping the course of an inflammatory response through production of large amounts of inflammatory mediators, including the proinflammatory cytokines TNF$\alpha$, IL-6, and IL-12, and the anti-inflammatory cytokine IL-10[31]. Activation of the $A_{2A}$AR on macrophages has been shown to suppress inflammatory responses by regulating proinflammatory cytokine production. For example, adenosine inhibits release of TNF$\alpha$ and IL-12 from macrophages induced by various stimuli, predominantly through activation of $A_{2A}$ARs[32,33], although the $A_{2B}$AR also appears to play a role in the absence of functional $A_{2A}$ARs[32]. There is conflicting evidence for the mechanisms behind these effects. Elevation of cAMP in RAW264.7 macrophages has been shown to inhibit LPS-induced production of TNF$\alpha$ and macrophage inflammatory protein (MIP-1) via a PKA-dependent, EPAC-independent mechanism[34]. However, in primary peritoneal macrophages, $A_{2A}$AR suppression of TNF$\alpha$ production induced by extracellular matrix components was EPAC dependent and unaffected by the PKA inhibitor H89[35]. Meanwhile, Kreckler et al.[36] found evidence for an alternative mechanism that is independent of both PKA and EPAC, but can be reversed by an inhibitor of serine/threonine phosphatases. In the studies by Wall et al.[34] and Scheibner et al.[35], the reduction in TNF$\alpha$ release was associated with reduced activation of the central proinflammatory nuclear factor (NF) $\kappa$B signalling pathway, which could explain why the $A_{2A}$AR can suppress cytokine production induced by multiple stimuli. Wall et al.[34] also showed that PKA colocalises with the NF$\kappa$B inhibitory protein (I$\kappa$B) p105 on the A-kinase anchoring protein (AKAP) 95. This appears to facilitate PKA-mediated phosphorylation of p105 on a site that prevents its phosphorylation by I$\kappa$B kinases (IKKs), which is required for activation of NF$\kappa$B.

In addition to inhibiting proinflammatory cytokine production by macrophages, $A_{2A}$AR activation also promotes release of the anti-inflammatory cytokine IL-10. IL-10 protects against inflammatory tissue damage and autoimmunity by suppressing exaggerated responses in several different cell types. For example, in macrophages, it inhibits production of proinflammatory cytokines such as IL-6, IL-12, and TNFα, and chemokines including monocyte chemotactic protein 1 (MCP1), IL-8, and MIP-2, and also inhibits antigen presentation and T-cell stimulatory capacity by suppressing MHCII and costimulatory molecule expression. Meanwhile in $CD4^+$ T cells, IL-10 inhibits proliferation and production of cytokines such as IL-2, IFNγ, and TNFα, and is thought to contribute to the suppressive activity of regulatory T cells[37,38].

In primary human and murine monocytes and in monocytic cell lines, CGS21680 potentiates LPS-induced IL-10 production[39,40,41]. Meanwhile, in macrophages from $A_{2A}$AR-deficient mice, lack of a functional $A_{2A}$AR blocks IL-10 production[41]. Studies in RAW264.7 macrophages have shown that C/EBPβ is the major transcription factor regulating the stimulatory effect of adenosine on *E. coli*-induced IL-10 production[41]. Interestingly, LPS induction of IL-10 production in this cell type has been show to be PKA independent[42]. In another study, PKA-independent activation of C/EBPβ by EPAC1 was found to regulate expression of the anti-inflammatory protein SOCS3[43]. It may be that EPAC1 also regulates C/EBPβ-mediated IL-10 production in macrophages. The role of PKA was not addressed in the study by Csóka et al.[41]; however, a requirement for p38 MAPK activity was identified.

## T Cells

Under normal circumstances, the powerful innate mechanisms initiated by neutrophils and macrophages result in the effective elimination of pathogenic material and the subsequent resolution of the inflammatory response. However, in the case of chronic inflammatory disease, inflammation persists and the adaptive arm of the immune system is then engaged. Macrophages and dendritic cells migrate from the site of inflammation to the lymph nodes, where they present processed antigens to specific circulating naïve T cells. In the presence of the correct costimulatory signals, T cells are activated and differentiate into different classes of effector cells guided by the particular cytokine profile present. $CD4^+$ T cells are activated by antigens derived from phagocytosed material and may differentiate into one of several different classes of “helper” T cells, including Th1, Th2, or regulatory T cells (Tregs), which differ in their effector functions and the cytokines they produce. Th1 cells are characterised by the production of IFNγ, which potently activates macrophages and generally promotes cell-mediated immunity. This is important for pathogen clearance, but also promotes inflammation and associated tissue damage. Th2 cells make IL-4, IL-5, and IL-13, which are involved in humoral immunity directed against extracellular pathogens[44,45]. Cytotoxic $CD8^+$ T cells arise when T cells are activated by cells presenting intracellularly derived antigens rather than ingested material.

The $A_{2A}$AR has a suppressive effect on many aspects of T-cell function, inhibiting proinflammatory cytokine production by both $CD8^+$ and $CD4^+$ cells[46,47], and suppressing $CD8^+$ cytolytic activity[46]. $A_{2A}$AR activation also suppresses proliferation of developing and mature Th1 and Th2 $CD4^+$ cells by reducing expression of IL-2 and CD25, the α-subunit of the IL-2 receptor complex[47,48]. A reduction in the positive costimulatory molecule CD40L and an increase in expression of negative costimulatory molecules PD-1 and CTLA-4 has also been observed[47]. Inhibition of T-cell activation appears to be mediated at least in part by PKA as in the study by Sevigny et al.[47], H89 reversed the inhibitory effect of ATL313 on phosphorylation of ZAP70, a tyrosine kinase involved in downstream signalling from the TCR. Furthermore, suppression of T-cell cytokine production by CGS21680 can be mimicked by selective activation of PKA[46]. However, the kinase activity of PKA may not be important, as two catalytic site inhibitors (H89 and a PKA inhibitor peptide) could not inhibit the response, while an antagonist of the cAMP binding site on the regulatory subunit of PKA was effective[47].

$A_{2A}$AR activation also appears to be important in mediating the immunosuppressive effects of Tregs. Tregs are identified by their ability to suppress effector T-cell responses and by the expression of a defined set of molecular markers, including CD4, CD25, and the X chromosome–encoded forkhead transcription factor Foxp3[49]. However, Tregs can also be specifically differentiated from other T-cell subsets by the presence of high levels of CD39[50] and CD73[50,51]. Adenosine generated by these enzymes suppresses T-cell function by binding to $A_{2A}$ARs on target cells[50]. In addition, $A_{2A}$AR activation has been shown to promote the generation of Tregs by up-regulating Foxp3 expression[52].

Another small subset of T cells regulated by the $A_{2A}$AR is formed by the invariant natural killer T (iNKT) cells, which express an invariant TCR alongside NK cell markers such as NK1.1[4,53]. iNKT cells function in innate immunity by recognising glycolipid antigens presented on the MHC1-related molecule CD1d and can be rapidly activated early in inflammatory responses to produce copious quantities of cytokines[53]. iNKT cells express all four AR subtypes, but the $A_{2A}$AR is most prevalent[54]. Activation of the $A_{2A}$AR inhibits production of IFNγ induced by the marine sponge glycolipid α-galactoceramide[54,55]. In contrast, production of IL-4, IL-10, and TGFβ are enhanced[54], indicating that rather than having a nonbiased effect on cytokine production as is seen in conventional T-cell subtypes[48], $A_{2A}$AR activation skews the cytokine profile of activated iNKT cells away from a proinflammatory Th1 phenoptype towards a Th2 phenotype[54]. This has potentially important implications for the treatment of diseases such as rheumatoid arthritis and Crohn's disease, where cell-mediated Th1 responses drive the chronic inflammatory phenotype.

## General Mechanisms

The ability of $A_{2A}$AR signalling to suppress inflammatory responses in such a wide range of cell types and in response to diverse stimuli has raised the question of whether or not there are common mechanisms by which the receptor exerts its effects. One answer to this question is suggested by findings that the $A_{2A}$AR is able to dampen activation of two major proinflammatory signalling pathways: the NFκB and the janus kinase/signal transducer and activator of transcription (JAK/STAT) pathways. NFκB is a transcription factor activated by a wide variety of stimuli, including proinflammatory cytokines (such as TNFα and IL-1) and bacterial products (such as LPS)[56]. NFκB plays a pivotal role in the initiation and perpetuation of an immune response by triggering expression of major inflammatory mediators such as cytokines, chemokines, and adhesion molecules[57,58]. The suppressive effect of adenosine signalling on the NFκB pathway has been demonstrated in a range of cell types[59]. However, subsequent studies have revealed that the mechanisms behind the inhibitory effect differ between cell types.

In resting conditions, NFκB transcription factors, of which the p50/RelA dimeric complex is best characterised, reside in the cytoplasm in complex with the inhibitory protein IκB. Upon activation of the NFκB pathway by stimuli such as TNFα or LPS, IκBα is phosphorylated, polyubiquitinated, and degraded by the proteasome, thereby freeing NFκB to translocate to the nucleus to modulate transcription[60]. In a study using a human myeloid cell line, adenosine inhibited TNFα-induced NFκB DNA-binding activity and NFκB-dependent gene expression by inhibiting nuclear translocation of active NFκB without affecting IκBα phosphorylation or degradation[59]. The role of the $A_{2A}$AR was confirmed in a later study using macrophages from $A_{2A}$AR-deficient mice that displayed enhanced TLR-induced NFκB DNA binding compared to wild-type cells[61]. In these cells, this was accompanied by increased phosphorylation and accelerated degradation of IκBα, indicating that the $A_{2A}$AR was having effect at an earlier stage in the NFκB pathway. In agreement with these findings, Sands et al.[62] found that increasing expression of the $A_{2A}$AR in rat C6 glioma cells inhibited TNFα and LPS-induced NFκB DNA binding and expression of target genes, and again this was accompanied by a decrease in phosphorylation and degradation of IκBα. However, in the same study, increased $A_{2A}$AR expression suppressed NFκB activation in HUVECs without affecting phosphorylation or the degradation pattern of IκBα. These differing results highlight the cell-specific nature of the $A_{2A}$AR-mediated response.

One mechanism that may prevent activation of NFκB through inhibition of IκBα phosphorylation and degradation is suggested by a recent study in which adenosine was found to increase SUMO-1

modification of IκBα[63]. SUMO-1 is a small ubiquitin-like molecule that acts to restore levels of IκBα at the end of an NFκB-mediated response[64]. In a process similar to ubiquitination, SUMO-1 is conjugated to newly synthesised IκBα at the same $Lys^{21}/Lys^{22}$ ubiquitination site. This prevents its ubiquitination and degradation, thereby switching off the NFκB pathway[65]. Interestingly, in HeLa cells subjected to hypoxia, prior exposure to cycles of hypoxia/reoxygenation to induce adenosine accumulation suppressed NFκB activation with a parallel increase in levels of SUMO-1 conjugated to IκBα[63]. Meanwhile, phosphorylation of IκBα was not observed. Although the precise receptor involved in generating the response was not identified, the nonselective $A_2AR$ agonist NECA also increased SUMO-conjugated IκBα in a dose-dependent manner and this could only be partially inhibited by the $A_{2B}AR$ antagonist PBS 1115, suggesting that this may be a mechanism relevant to $A_{2A}AR$-mediated suppression of the NFκB pathway. This could explain the findings of Sands et al.[62] in C6 glioma cells. Overexpression of the $A_{2A}AR$ may lead to increased SUMOylation of IκBα, which would block its polyubiquitination, leading to the observed inhibition of NFκB activity.

An alternative mechanism by which adenosine can inhibit NFκB activation is by directly interfering with ubiquitination of IκBα by the IκBα-specific E3 SCF Ub ligase complex[66]. This complex is composed of Skp1, Cul-1, and β-TrCP F-box subunits and its activation is dependent upon modification of the Cul-1 subunit by the ubiquitin-like molecule Nedd8[67]. Adenosine produced during hypoxic preconditioning has been found to promote removal of Nedd8 from the Cul-1 subunit, thereby inactivating the complex and preventing IκBα ubiquitination and degradation[66]. The contribution of different adenosine receptors to this response has not been fully investigated, but if the $A_{2A}AR$ is involved, then the absence of this mechanism might explain the accelerated degradation of IκBα observed in $A_{2A}AR$-deficient mice[61].

Another generalised mechanism by which the $A_{2A}AR$ may be able to suppress inflammation in a number of cell types is through inhibition of the JAK/STAT pathway. This pathway transduces signals from receptors for the haemopoietin group of cytokines that includes the IL-6 family. Manipulation of the IL-6 response may be beneficial in inflammatory disease, as IL-6 is a key regulator of the immune response with a wide spectrum of activities including regulation of haematopoeisis, induction of inflammation, and generation of the acute phase response[68]. In addition, IL-6 family cytokines are major participants in the pathology of diseases such as atherosclerosis[69], rheumatoid arthritis[70], and inflammatory bowel disease[71]. The IL-6 receptor is composed of an IL-6–binding subunit and a signal-transducing subunit (gp130) that is common to all IL-6–type cytokines. Following activation of the receptor, associated JAKs become activated and phosphorylate specific tyrosine residues on the gp130 subunit. STAT proteins are recruited to the phosphorylated receptor, where they also become tyrosine phosphorylated and activated, allowing them to form dimers that translocate to the nucleus to modulate transcription of IL-6–regulated genes such as vascular endothelial growth factor, cyclin D1, and $BclX_L$ (Fig. 2)[72,73].

Signalling from the $A_{2A}AR$ has been found to inhibit JAK/STAT cytokine signalling through receptors of the gp130 family by inducing expression of the anti-inflammatory protein SOCS3[74]. SOCS proteins are characterised by a central SH2 domain, an N-terminal kinase inhibitory region (KIR), and a C-terminal "SOCS box" domain[75]. They mediate their inhibitory effects in part by binding to phosphorylated tyrosine residues on activated receptors and interacting with receptor-associated JAKs via the KIR[72]. This inhibits JAK activity and prevents phosphorylation and activation of STAT proteins, thereby terminating signalling (Fig. 2)[75]. In endothelial cells, MEFs, and COS-1 cells, cAMP-induced SOCS3 expression inhibits IL-6–stimulated STAT3 phosphorylation via a PKA-independent EPAC1-mediated mechanism[74,76]. This relies upon activation of Rap1 by EPAC1 and involves CAAT/enhancer-binding protein (C/EBP) transcription factors, namely C/EBPβ and C/EBPδ[43]. The steps linking EPAC1/Rap1 activation to SOCS3 expression are not well defined, but there is a requirement for ERK-mediated phosphorylation of C/EBPβ on $Thr^{235}$[76]. This may be a priming step and other pathways are likely to be involved, as EPAC1 depletion in HUVECs does not block ERK activation[77]. A role for a PKC-mediated pathway has been suggested by studies in COS-1 cells in which down-regulation of PLCε or PKCα activity blocked cAMP-induced SOCS3 expression[76].

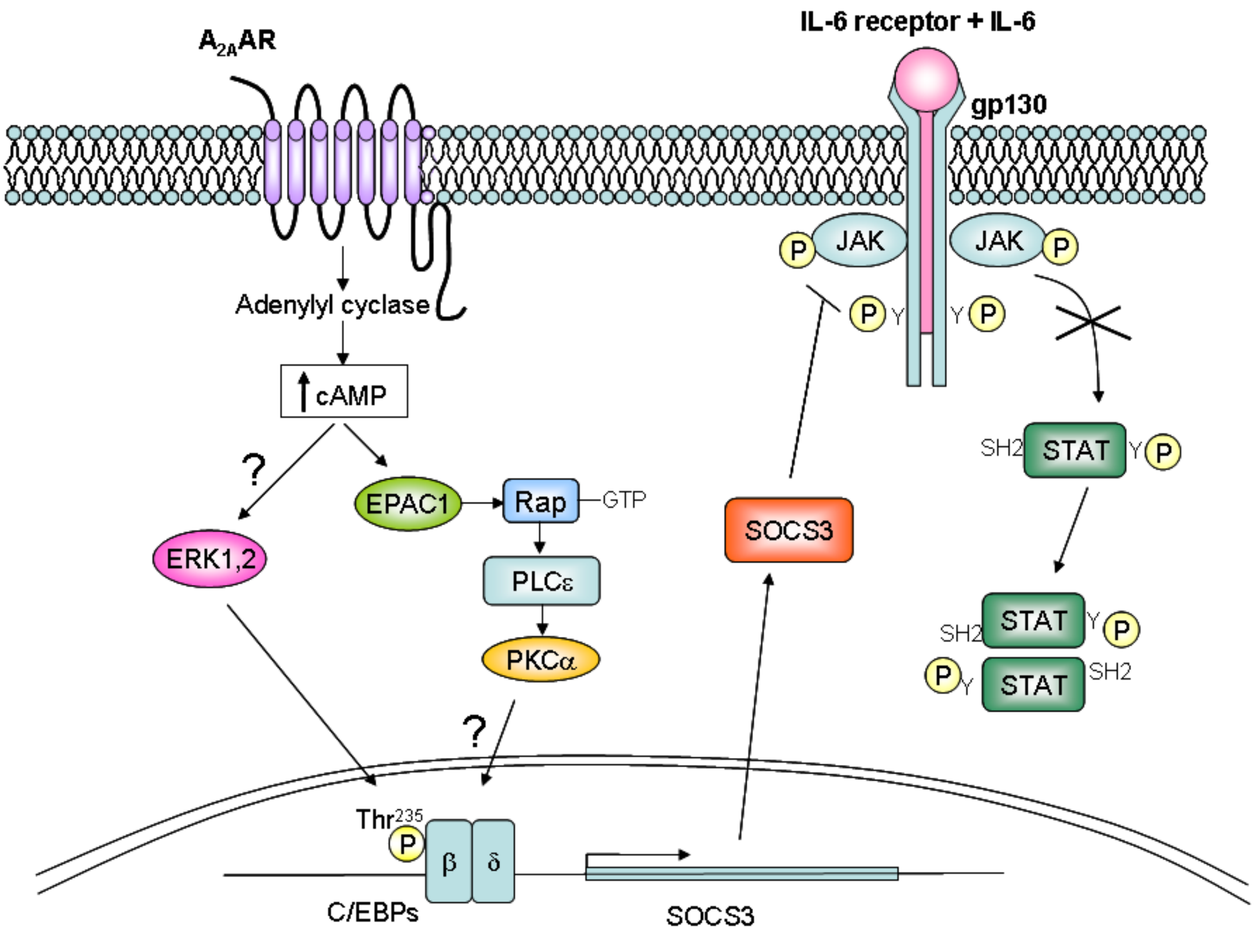

**FIGURE 2.** Activation of the $A_{2A}$AR suppresses cytokine-mediated JAK/STAT signalling via induction of the inhibitory protein SOCS3. Upon binding of adenosine or synthetic agonists to the $A_{2A}$AR, Gs stimulation of adenylyl cyclase results in an elevation of intracellular cAMP levels and activation of cAMP effectors, including EPAC1. EPAC1 acts as a guanine nucleotide exchange factor for Rap1, causing Rap1-GTP to accumulate. This leads to activation of PLCε and hydrolysis of phosphatidylinositol-4,5-bisphosphate to generate inositol-1,4,5-trisphosphate ($IP_3$) and *sn*-1,2-diacylglycerol (DAG). $IP_3$ mobilises $Ca^{2+}$ from intracellular stores, which then stimulates a further increase in cytosolic $Ca^{2+}$ by activation of store-operated $Ca^{2+}$ channels on the plasma membrane. The cytosolic $Ca^{2+}$ and DAG generated from these events bind and activate PKCα, which appears to be important for SOCS3 expression. Elevation of cAMP also results in activation of the ERK pathway and ERK-mediated phosphorylation of C/EBPβ on Thr235, which is required for induction of SOCS3. Newly synthesised SOCS3 interacts with Tyr-phosphorylated gp130 subunits of IL-6 family cytokine receptors and inhibits JAK activity, thereby preventing STAT phosphorylation on Tyr705 and its subsequent activation.

Recently, another mechanism by which the $A_{2A}$AR can negatively regulate the JAK/STAT pathway has been identified. In endothelial cells stimulated with IL-6, overexpression of the $A_{2A}$AR suppressed downstream signalling by inducing polyubiquitination and proteasomal degradation of STAT proteins[78]. This required prior activation of STAT3 by cytokine-directed JAK-mediated phosphorylation on $Tyr^{705}$. This may act as a signal for ubiquitination, or since activation of STATs induces their nuclear translocation, it may be required to colocalise STAT3 with a specific, as yet unidentified E3 ligase.

## *IN VIVO* EVIDENCE FOR THE ANTI-INFLAMMATORY PROPERTIES OF THE $A_{2A}$AR

*In vitro* studies such as those discussed above provide an overwhelming amount of evidence to support the ability of $A_{2A}$AR signalling to inhibit the inflammatory actions of specific cell types. The anti-inflammatory and tissue-protective effects elicited by the $A_{2A}$AR have also been demonstrated *in vivo* in numerous studies using animal models of inflammatory disease. These studies have centred on assessing the ability of $A_{2A}$AR agonists to protect against excessive inflammation and tissue injury or, conversely, on the effects of either $A_{2A}$AR antagonism or gene deletion in exacerbating inflammatory damage.

### Liver

The ability of the $A_{2A}$AR to offer protection against excessive inflammation and tissue injury in the liver has been demonstrated in a number of studies using the hepatotoxic T-lymphocyte activator concanavalin A (ConA)[79,80,81]. In mouse models, intravenous administration of ConA induces liver damage, which is dependent upon activation of $CD4^+$ T cells by macrophages[82] and subsequent release of proinflammatory cytokines such as TNF$\alpha$, IL-6, and IFN$\gamma$[80,83,84]. In mice, administration of the $A_{2A}$AR-selective agonists CGS21680[79] or ATL146e[80] reduced ConA-induced liver injury and suppressed proinflammatory cytokine production, which is consistent with the ability of $A_{2A}$AR activation to suppress T-cell activation[46,47] and TNF$\alpha$ production by macrophages *in vitro*[32,33]. Conversely, treatment with the $A_{2A}$AR antagonist ZM241385 exacerbated liver injury and promoted cytokine release in response to ConA[79]. The protective nature of $A_{2A}$AR activation was confirmed in $A_{2A}$AR-deficient mice, where treatment with ConA caused drastically enhanced liver damage and cytokine production compared to wild-type mice[79].

The role of the $A_{2A}$AR in regulating inflammatory responses in the liver has also been well studied in models of ischaemia reperfusion injury (IRI). IRI refers to the damage caused to tissues by the restoration of blood flow following a period of restricted blood flow and oxygen deprivation. What follows initially is an innate immune system–mediated proinflammatory reaction involving production of reactive oxygen species, proinflammatory cytokines, and chemokines, and the up-regulation of adhesion molecules on the endothelium. Large numbers of inflammatory cells then infiltrate the damaged organ, followed by T cells as the adaptive immune response is activated, leading to irreversible cell damage, vascular occlusion, and organ dysfunction[85,86,87].

Activation of the $A_{2A}$AR has been found to protect against inflammatory damage caused by ischaemia and reperfusion in a number of tissues including liver[88], kidney[89], lung[90], and heart[91]. For example, in mice subjected to hepatic IRI, the $A_{2A}$AR-selective agonist ATL146e caused a dramatic reduction in tissue injury that was associated with reduced neutrophil accumulation and cytokine and chemokine expression[88]. Meanwhile, in $A_{2A}$AR-deficient mice, liver injury was exacerbated compared with wild-type mice, indicating that activation of $A_{2A}$ARs by endogenously produced adenosine has a similar effect[88]. Through the use of chimaeric mice, it was demonstrated that protection occurred via activation of $A_{2A}$ARs on bone marrow–derived cells (BMDCs), as agonists were effective in reducing IRI in mice expressing $A_{2A}$ARs specifically on BMDCs, but not in mice selectively lacking $A_{2A}$ARs on BMDCs[92]. Subsequently, the beneficial effects of $A_{2A}$AR agonists in IRI were linked to activation of $A_{2A}$ARs on iNKT cells. Lappas et al.[93] found that similarly to mice treated with ATL146e, protection against IRI was also observed in RAG-1-deficient mice that lack mature lymphocytes and in mice in which iNKT cells had been depleted or inactivated. Meanwhile, liver injury in RAG-1–deficient mice could be reconstituted to wild-type levels by adoptive transfer of $NK1.1^+$ cells from wild-type mice. These findings suggested that iNKT cells play a critical role in mediating IRI. Adoptive transfer of NK cells from $A_{2A}$-deficient mice also reconstituted liver injury in RAG-1–deficient mice, but IRI could not be attenuated by ATL146e, indicating that ATL146e exerts its effects by activating receptors on NKT cells[93].

Activation of the $A_{2A}$AR has also proved beneficial in protecting against IRI in rats receiving partial liver transplants. Treatment with CGS21680 for 3 h immediately after reperfusion of the transplanted liver dramatically increased survival rate and improved liver function. This was associated with decreased neutrophil infiltration and inhibition of TNFα, IL-1β, and IL-6 expression[94]. Further investigation of the mechanisms involved revealed that CGS21680 inhibited IKK-mediated phosphorylation and subsequent NFκB activation in liver, and reduced expression of major κB-regulated genes, such as MIP-1, ICAM-1, and TNFα, while the $A_{2A}$AR-selective antagonist ZM241385 had the opposite effect[95].

## Kidney

$A_{2A}$AR agonists also reduce tissue injury in animal models of renal IRI[89,96,97]. This has been linked to inhibition of neutrophil adhesion to the endothelium. Okusa et al.[96] found that when rats were treated with ATL146e, reduced IRI could be correlated with an inhibition of endothelial P-selectin and ICAM-1 expression and decreased neutrophil infiltration. As in the liver, activation of $A_{2A}$ARs on BMDCs appears to be responsible for this effect, as ATL146e did not protect against IRI in chimaeric mice lacking $A_{2A}$ARs on BMDCs, but was effective in mice expressing the $A_{2A}$AR only on BMDCs[97]. A role for macrophages in mediating renal IRI mice has been demonstrated in experiments where depletion of macrophages reduced injury[92]. Injury could be reconstituted by adoptive transfer of RAW264.7 macrophages, an effect which was inhibited by treatment with ATL146e[92]. However, the protective effect was not mediated via direct activation of receptors on macrophages, as ATL146e was equally effective in reducing injury in macrophage-depleted mice reconstituted with wild-type or $A_{2A}$AR-deficient macrophages, and had no effect in $A_{2A}$AR-deficient mice even when reconstituted with wild-type macrophages[92]. Further investigation using RAG-1 knock-out mice has revealed the role of $A_{2A}$ARs on $CD4^+$ T cells in mediating protection against renal IRI[98]. These mice suffered reduced IRI compared to wild-type mice, but injury was restored by adoptive transfer of wild-type or $A_{2A}$AR knock-out $CD4^+$ T cells. ATL146e reduced injury through activation of receptors on $CD4^+$ T cells, as it was only effective in mice reconstituted with wild-type $CD4^+$ T cells and not those lacking the $A_{2A}$AR[98].

While the protective effects of $A_{2A}$AR agonists in renal IRI are not mediated by receptors on macrophages, these cells do appear to be important in preventing renal injury in experimentally induced glomerulonephritis[99]. In one study, treatment of rats with CGS21680 reduced kidney injury in the acute inflammatory and chronic stages of disease, and decreased infiltration of $CD8^+$ T cells and macrophages. This was attributed to effects on macrophages, as $A_{2A}$AR expression increased in macrophages following disease induction, while expression on $CD8^+$ T cells remained unchanged. In addition, renal protection was associated with reduced expression of macrophage-derived chemokines and increased production of the anti-inflammatory cytokines IL-4 and IL-10. Meanwhile, nonmacrophage-derived chemokines were not affected[99]. Macrophages are also thought to be important for the protective effect of $A_{2A}$AR activation on kidney function in rats subjected to streptozotocin-induced diabetes. Awad et al.[100] found that ATL146e reduced renal injury and inhibited glomerular infiltration by macrophages, which are thought to be major contributors to the development of renal failure in diabetes. This was associated with decreased expression of proinflammatory cytokines TNFα and IFNγ, which are important predictors of diabetic nephropathy[100].

## Heart

In the heart, $A_{2A}$AR agonists have been shown to suppress IRI-associated inflammatory events and reduce myocardial infarct size in a number of animal models, including rabbits[101], dogs[91], mice[102], and pigs[103]. Several studies have focussed on the effect of $A_{2A}$AR stimulation on the activity of neutrophils, which are thought to be major perpetrators of IRI in the heart[104]. For example, in a canine model of reperfused myocardial infarction, ATL146e administered as either a pretreatment, or 30 min before and continuing through reperfusion, caused a reduction in infarct size that was correlated with an inhibition of neutrophil accumulation and activity as measured by suppression of P-selectin expression[91]. In a similar study, CGS21680 administered 5 min before reperfusion reduced IRI and inhibited neutrophil superoxide generation and endothelial adherence[105]. However, more recent studies indicate that suppression of neutrophil activity by $A_{2A}$AR agonists may be secondary to their effects on T cells. In a study using $A_{2A}$AR knock-out mice in combination with bone marrow transplantation, ATL146e reduced infarct size and associated neutrophil and T-cell accumulation only when the wild-type receptor was expressed in bone marrow[102]. Infarct size and neutrophil infiltration were also reduced in RAG-1 knock-out mice, but this could not be enhanced by ATL146e, indicating that $A_{2A}$ARs on lymphocytes mediate the protective effects of the agonist[102]. In a subsequent study, it was found that injury to RAG-1 knock-out mice could be restored by adoptive transfer of $CD4^+$ T cells from wild-type mice, but not $CD8^+$ T cells from wild-type mice or $CD4^+$ T cells from IFNγ knock-out mice[106]. Meanwhile, ATL146e blocked restoration of injury in mice receiving $CD4^+$ T cells from wild-type mice, but not $A_{2A}$AR knock-out mice, indicating that $A_{2A}$ARs on $CD4^+$ T cells are responsible for the beneficial effects of $A_{2A}$AR agonists in myocardial IRI[106].

## Lung

The ability of $A_{2A}$AR agonists to reduce lung inflammation has been demonstrated in a number of animal models of asthma and other COPDs. For example, intratracheal administration of CGS21680 inhibited pulmonary inflammation induced by ovalbumin challenge in sensitised brown Norwegian rats as indicated by a decrease in neutrophil and eosinophil numbers in bronchoalveolar lavage fluid (BALF)[107]. Similar findings were produced in ovalbumin-sensitised mice, where CGS21680 treatment reduced lung infiltration of neutrophils, eosinophils, macrophages, and lymphocytes[108]. In contrast to these findings, CGS21680 had no effect on neutrophil influx in LPS or cigarette smoke–induced models of COPD, but it did reduce BALF levels of elastase in the LPS model, indicating a reduction in neutrophil activation[108].

$A_{2A}$AR-mediated suppression of lung inflammation has been further demonstrated using $A_{2A}$AR-deficient mice. Nadeem et al.[109] found that following allergen challenge of ragweed-sensitised mice, levels of neutrophils, eosinophils, and lymphocytes were significantly increased in BALF from $A_{2A}$AR-deficient mice compared to similarly treated wild-type mice. This study also highlighted a possible mechanism behind the protective effects of the $A_{2A}$AR in the lung. Sensitised mice lacking the $A_{2A}$AR expressed increased levels of the p65 NFκB subunit and phospho-IκBα compared to wild-type sensitised mice. This was associated with up-regulation of the NFκB-regulated gene inducible nitric oxide synthase (iNOS). These results indicate that the $A_{2A}$AR inhibits lung inflammation at least in part by suppressing NFκB activation and downstream gene expression[109].

$A_{2A}$AR agonists play a similarly protective role in models of acute respiratory distress syndrome (ARDS). In fact, the critical nature of the hypoxia/adenosine/$A_{2A}$AR axis in the lung has been demonstrated in a study investigating the adverse effects of oxygen therapy in ARDS. Thiel et al.[110] found that life-saving oxygen therapy exacerbated inflammatory lung damage in mice subjected to polymicrobial lung infection. Damage was severely enhanced in $A_{2A}$AR-deficient mice and in wild-type mice treated with the $A_{2A}$AR antagonist ZM241385. Meanwhile, treatment with CGS21680 reduced lung damage and dramatically improved survival rates of oxygenated wild-type mice, confirming the role of the $A_{2A}$AR in hypoxia-induced protection[110]. In another study using LPS treatment to produce ARDS-like symptoms, the $A_{2A}$AR-selective agonist ATL202 inhibited neutrophil recruitment and suppressed levels of IL-6, TNFα, and the chemotactic molecules keratinocyte-derived chemoattractant (KC) and MIP-2 in the lung[111]. Again, $A_{2A}$AR-deficient mice experienced exaggerated responses to LPS, which could not be attenuated by ATL202. Inhibition of lung inflammation by ATL202 was attributed to activation of $A_{2A}$ARs expressed on haemopoietic cells, as the agonist was effective in chimaeric mice only when the $A_{2A}$AR was present on BMDCs[111].

As in other tissues, $A_{2A}$AR activation protects against inflammatory tissue damage in the lung caused by IRI[90,112,113]. In a rat model of global ischaemia induced by trauma and haemorrhagic shock, CGS21680 reduced neutrophil infiltration and activation and attenuated lung injury[112]. Evidence for the specific cell types involved in this $A_{2A}$AR-mediated response has been obtained using a mouse model of lung IRI in which ATL313 inhibited lung injury and reduced $CD4^+$ T-cell activation and neutrophil recruitment[90]. Antibody depletion of neutrophils or $CD4^+$ T cells also reduced lung injury, highlighting the key role that these cell types play in lung IRI. ATL313 treatment offered no further protection in this case, indicating that the anti-inflammatory effects of the agonist are achieved through effects on $CD4^+$ T cells and neutrophils. Levels of inflammatory cytokines and chemokines (TNFα, IL-17, KC, MCP-1, MIP-1, and RANTES) in BALF were also lower in the absence of neutrophils or $CD4^+$ T cells, but could only be further reduced by ATL313 in neutrophil-depleted mice, indicating that suppression of neutrophil activity may be secondary to ligation of $A_{2A}$ARs on $CD4^+$ T cells[90].

In addition to suppressing the activity of proinflammatory cells, *in vitro* studies have shown that activation of the $A_{2A}$AR can also promote the development of Treg cells[52]. This is supported by *in vivo* data from a study of a murine model of autoimmune pneumonitis induced by adoptive transfer of self-reactive T cells. CGS21680 reduced lung inflammation and fatalities from autoimmune pneumonitis by promoting T-cell anergy and production of $Foxp3^+$ and $LAG3^+$ Tregs[52].

## Vasculature

$A_{2A}$AR activation has proven beneficial in the injured vasculature, inhibiting inflammatory events that lead to arterial lesion formation and the development of atherosclerosis. For example, in the murine carotid artery ligation model of arterial inflammation, lesion formation was markedly reduced in mice treated with the $A_{2A}$AR agonist ATL146e. This was attributed to an inhibition of neutrophil and macrophage recruitment via suppression of VCAM-1, ICAM-1, and P-selectin expression[114].

The importance of $A_{2A}$AR activation by endogenously produced adenosine has also been explored using mice lacking the ecto-5'-nucleotidase CD73, which catalyses the extracellular conversion of 5'-AMP to adenosine[115]. Following wire-induced injury of the carotid artery, these mice experienced increased neointimal lesion formation compared to wild-type mice, which was associated with elevated VCAM-1 expression, monocyte arrest, and NFκB activation. This was determined to be due to an absence of the adenosine/$A_{2A}$AR pathway, as VCAM-1 expression and levels of monocyte adhesion in cultured $CD73^{-/-}$ endothelial cells were reduced to wild-type levels by ATL146e. These findings were supported by *in vivo* studies in which ATL146e suppressed lesion formation in wild-type mice subjected to wire injury and reduced it to wild-type levels in uninjured $CD73^{-/-}$ mice[115].

The suppression of adhesion molecule expression reported in these studies is in keeping with *in vitro* data describing effects of the $A_{2A}$AR on adhesion molecule expression and inflammatory responses in neutrophils and endothelial cells[30,62]. However, in another study using mice engineered to be both $A_{2A}$AR deficient and atherosclerosis prone ($A_{2A}AR^{-/-}/ApoE^{-/-}$), wire injury to the carotid artery induced enhanced lesion formation compared to wild-type/$ApoE^{-/-}$ mice without affecting levels of homing molecules on neutrophils or endothelial cells[116]. Indeed, endothelial cells were not deemed to be involved, as the level of neointimal injury in chimaeric $A_{2A}AR^{+/+}/ApoE^{-/-}$ mice lacking $A_{2A}$ARs on BMDCs was similar to that of the whole-animal knock-out, while injury in $A_{2A}AR^{-/-}/ApoE^{-/-}$ animals was reduced to wild-type levels by bone marrow transplant from $A_{2A}AR^{+/+}/ApoE^{-/-}$ mice. Instead, increased neutrophil rolling and adhesion observed was attributed to potentiation of PSGL-1 clustering on the neutrophil surface and heightened affinity of $\beta_2$ integrins[116].

This story is further complicated by results from an earlier study by the same group in which $A_{2A}$AR deficiency was determined to be protective against development of atherosclerotic lesions in $ApoE^{-/-}$ mice[117]. Although these mice had much higher IL-1β and IL-6 levels and enhanced NFκB activation in lesions compared to $A_{2A}AR^{+/+}/ApoE^{-/-}$ mice, the number of macrophages and foam cells present, and therefore lesion size, was greatly reduced. However, this effect may be specific to this particular model as in other studies, $A_{2A}$AR activation has been found to inhibit foam cell formation in murine macrophages through stimulation of cholesterol efflux[118,119].

## Brain

The role of the $A_{2A}$AR in regulating tissue injury in the brain is complex. In a number of animal models, administration of agonists has proven beneficial, reducing neuronal cell damage caused by kainate-induced excitotoxicity[120], haemorrhagic stroke[121], and forebrain ischemia[122], and improving neurological outcome following ischemic or traumatic spinal cord injury[123,124,125]. Paradoxically, in many cases, inactivation of the receptor also provides protection against brain injury. For example, ZM241385 reduced kainate-induced damage in rats, while the selective $A_{2A}$AR antagonist SCH 58261 reduced brain injury in a rat model of focal cerebral ischemia[126,127]. Meanwhile, genetic deficiency of the $A_{2A}$AR reduces brain damage following ischemic[128,129,130] or traumatic brain injury[131,132].

As in other tissues, the beneficial effects of $A_{2A}$AR agonists appear to be associated with a reduction in inflammation. For example, in rats subjected to haemorrhagic stroke, administration of CGS21680 to the striatum inhibited TNF$\alpha$ production and neutrophil infiltration and reduced cell death[121]. Meanwhile, the improved neurological outcome observed in rabbits treated with ATL146e following spinal cord trauma was associated with reduced infiltration of inflammatory cells[123].

The neuroprotective effects of $A_{2A}$AR antagonism or inactivation have been attributed to inhibition of other actions of $A_{2A}$AR in the brain, in particular $A_{2A}$AR-mediated promotion of glutamate release from neurons and glial cells and the subsequent excitotoxic cascade[129,130,133,134,135]. However, studies using mice either expressing or lacking the $A_{2A}$AR specifically in BMDCs showed that inflammatory cells are also involved[129]. In this study, selective inactivation of the $A_{2A}$AR on BMDCs attenuated ischaemic brain injury following middle cerebral artery occlusion. This was associated with reduced expression of the proinflammatory cytokines IL-12, IL-6, and IL-1, and an increase in the anti-inflammatory cytokine IL-10, suggesting that in this context, the presence of the $A_{2A}$AR is proinflammatory. This is in striking contrast to the effects observed in the liver where $A_{2A}$AR deficiency exacerbated ischemic injury, indicating that the nature of $A_{2A}$AR activity varies significantly between the brain and other tissues[129].

Further investigation has provided evidence that the inconsistent effects of $A_{2A}$AR activation or inactivation in the brain may occur as a result of fluctuations in the local levels of glutamate. In cultured glial cells, treatment with CGS21680 in the presence of low levels of glutamate increased intracellular cAMP levels and inhibited LPS-induced NOS activity in a PKA-dependent manner[136]. Meanwhile, at higher levels of glutamate, CGS21680 had no effect on cAMP levels, but promoted NOS activity via a PKC-dependent mechanism, prompting the authors to suggest that glutamate switches $A_{2A}$AR signalling from a PKA-dependent anti-inflammatory pathway to an alternative proinflammatory pathway. In support of these findings, CGS21680 administered to mice at times following traumatic brain injury, when glutamate levels were low, reduced brain injury and inhibited TNF$\alpha$ and IL-1 production, while treatment at times when glutamate levels were raised had the opposite effect[136].

## CONCLUSION/CLINICAL APPLICATIONS

The maintenance of normal tissue architecture and biological function requires successful resolution of inflammatory and immune responses following the clearance of infectious agents and completion of repair. This requires that proinflammatory responses be transient in nature, thus facilitating clearance of infection and initiating damage repair processes without proceeding to a chronic inflammatory state that ultimately leads to disease. An important determinant of the transience of proinflammatory processes is the existence of multiple endogenous proresolving and anti-inflammatory signalling pathways that actively turn off potentially damaging sustained inflammatory responses. These include lipid mediators, such as the resolvins and protectins, and annexin A1, amongst others[137,138]. As more details emerge on the molecular mechanisms responsible for the generation of such mediators and how they exert their protective effects, it is anticipated that their exploitation will be translated into new therapeutics to treat chronic inflammatory conditions[138].

As detailed above, there is now a vast body of evidence, both at the cellular level and in disease models in whole animals, pointing toward the adenosine/$A_{2A}$AR signalling module functioning similarly as an endogenous protective anti-inflammatory and immunosuppressive system. Therefore, as has been argued for the annexin A1 system[138], exploiting endogenous protective pathways pharmacologically by the development of $A_{2A}$AR-selective agonists may be useful as treatments for conditions where inappropriate proinflammatory responses drive tissue damage and inflammatory disease. Moreover, by simply mimicking an endogenous protective pathway, it would be anticipated that side effects arising from $A_{2A}$AR-selective agonist administration would be minimal.

In support of this argument, several $A_{2A}$AR agonists have been approved for clinical trials based on their anti-inflammatory and wound-healing properties[139]. For example, topical application of the $A_{2A}$AR-selective agonist MRE-0094 to promote healing of diabetic foot ulcers has recently been tested in phase II clinical trials[140]. $A_{2A}$AR agonists have also been tested for their suitability as pharmacological stress agents for use in myocardial perfusion imaging because of their vasodilatory properties. ATL146e and two other $A_{2A}$AR-selective agonists (MRE0470 and Reganodenoson) were proven in phase III trials to be as effective as adenosine, which is currently used in the clinic, but produced fewer side effects[139]. Owing to the wide tissue distribution of $A_{2A}$ARs, side effects of $A_{2A}$AR agonists themselves also present a problem when considering long-term treatment for chronic inflammatory conditions. The $A_{2A}$AR regulates blood pressure and platelet aggregation, and has a variety of effects in the central nervous system, including interactions with the $D_2$ dopamine receptor[141,142]. Finally, while hypotension has been observed in animals treated with CGS21680[107,112], this may be avoidable through use of more selective agonists, such as ATL146e, which has a more favourable therapeutic window, as it induces anti-inflammatory responses at concentrations much lower than those required to activate haemodynamic effects[7].

The adenosine/$A_{2A}$AR axis has evolved as an invaluable endogenous response to protect against overzealous actions of the immune system. A large number of studies has demonstrated the ability of synthetic $A_{2A}$AR agonists to inhibit inflammatory responses and tissue damage in animal models. Findings from clinical trials currently in process will determine whether any of these agonists can be used safely and effectively to treat patients with inflammatory conditions. Meanwhile, further investigation of the molecular mechanisms triggered by $A_{2A}$AR activation will increase understanding of its actions in different tissues and may allow development of more targeted drugs to harness specific effects of $A_{2A}$AR signalling on different pathways without activating unwanted side effects.

## ACKNOWLEDGEMENTS

Work in TMP's laboratory is supported by the British Heart Foundation, Chest Heart and Stroke Scotland, and the U.K. Biotechnology and Biological Sciences Research Council.
